# Spatial analysis of hepatobiliary abnormalities in a population at high-risk of cholangiocarcinoma in Thailand

**DOI:** 10.1038/s41598-020-73771-0

**Published:** 2020-10-08

**Authors:** Kavin Thinkhamrop, Apiporn T. Suwannatrai, Nittaya Chamadol, Narong Khuntikeo, Bandit Thinkhamrop, Pongdech Sarakarn, Darren J. Gray, Kinley Wangdi, Archie C. A. Clements, Matthew Kelly

**Affiliations:** 1 Cholangiocarcinoma Screening and Care Program (CASCAP), Faculty of Medicine, Khon Kaen University, Khon Kaen, Thailand; 2 Data Management and Statistical Analysis Center (DAMASAC), Faculty of Public Health, Khon Kaen University, Khon Kaen, Thailand; 3 Health and Epidemiology Geoinformatics Research (HEGER), Faculty of Public Health, Khon Kaen University, Khon Kaen, Thailand; 4 Department of Parasitology, Faculty of Medicine, Khon Kaen University, Khon Kaen, Thailand; 5 Department of Radiology, Faculty of Medicine, Khon Kaen University, Khon Kaen, Thailand; 6 Department of Surgery, Faculty of Medicine, Khon Kaen University, Khon Kaen, Thailand; 7 Epidemiology and Biostatistics Section, Faculty of Public Health, Khon Kaen University, Khon Kaen, Thailand; 8 Department of Global Health, Research School of Population Health, Australian National University, Canberra, Australia; 9 Faculty of Health Sciences, Curtin University, Bentley, WA Australia; 10 Telethon Kids Institute, Nedlands, WA Australia

**Keywords:** Cancer, Diseases

## Abstract

Cholangiocarcinoma **(**CCA) is a serious health challenge with low survival prognosis. The liver fluke, *Opisthorchis viverrini*, plays a role in the aetiology of CCA, through hepatobiliary abnormalities: liver mass (LM), bile duct dilation, and periductal fibrosis (PDF). A population-based CCA screening program, the Cholangiocarcinoma Screening and Care Program, operates in Northeast Thailand. Hepatobiliary abnormalities were identified through ultrasonography. A multivariate zero-inflated, Poisson regression model measured associations between hepatobiliary abnormalities and covariates including age, sex, distance to water resource, and history of *O. viverrini* infection. Geographic distribution was described using Bayesian spatial analysis methods. Hepatobiliary abnormality prevalence was 38.7%; highest in males aged > 60 years (39.8%). PDF was most prevalent (20.1% of males). The Standardized Morbidity Ratio (SMR) for hepatobiliary abnormalities was highest in the lower and upper parts of the Northeast region. Hepatobiliary abnormalities specifically associated with CCA were also more common in males and those aged over 60 years and distributed along the Chi, Mun, and Songkram Rivers. Our findings demonstrated a high risk of hepatobiliary disorders in Northeast Thailand, likely associated with infection caused by *O. viverrini*. Screening for CCA and improvement of healthcare facilities to provide better treatment for CCA patients should be prioritized in these high-risk areas.

## Introduction

Southeast Asia is a high-risk area for liver cancer, including cholangiocarcinoma (CCA)^1^. Within Southeast Asia, the Northeast region of Thailand has the highest incidence of CCA, with an annual incidence of around 22.9 per 100,000 population, and with CCA being responsible for more than 60% of liver tumors in the region^2^. This burden persists despite frequent large-scale health education campaigns regarding the risk factors and behaviors that lead to the disease^3^. CCA is a particularly fatal cancer with few treatment options once the disease is in an advanced stage. It is therefore essential to identify early warning indications of the development of the disease so that a timely treatment plan can be put in place. As CCA is characterized by mass formation within the bile duct^3^, evidence of liver and biliary system disorders might provide an early indicator.

Liver and biliary system disorders preceding CCA in the human body comprise fatty liver disease (FLD), periductal fibrosis (PDF), cirrhosis, liver mass (LM), and bile duct dilatation (BDD). These abdominal abnormalities can be diagnosed by ultrasonography (US), which is widely used for this purpose^4,5^. These disorders are also associated with the progression of serious diseases, including liver cancer and CCA^6–16^. In the past, PDF has been detected in patients with CCA in areas endemic for the human liver fluke, *Opisthorchis viverrini*^17^. This liver fluke is a fish-borne trematode species. Human become infected by consuming raw or undercooked cyprinid fish that contain the infective agent. There are an estimated 10 million human *O. viverrini* infections in the Lao PDR, Cambodia, Myanmar, Vietnam, and Thailand, with particularly high prevalence in rural parts of Northeast Thailand^18^.

Given the high prevalence of liver and biliary tract abnormalities in Northeast Thailand (largely associated with the distribution of *O. viverrini* infection), detailed data on their geographic distribution is needed in order to identify high risk areas, and to inform health system programs and responses, particularly in terms of prevention of progression to CCA. Geographic information system (GIS) analysis has become a widely used tool in epidemiological research, allowing the identification of clustering and spatial patterns of disease, as well as the analysis of interactions between environmental factors and health. However, the authors are not aware of any studies that have used these methods in the study of the distribution of liver and biliary system abnormalities. This study used Bayesian spatial analysis methods to estimate prevalence rates and describe the distribution of hepatobiliary abnormalities in Northeast Thailand. This region is an endemic area for *O. viverrini* which is the main cause of abnormalities in the biliary system analysed here and which lies on the aetiological pathway to development of CCA^19^.

## Materials and methods

### Study area

**Figure 1:**
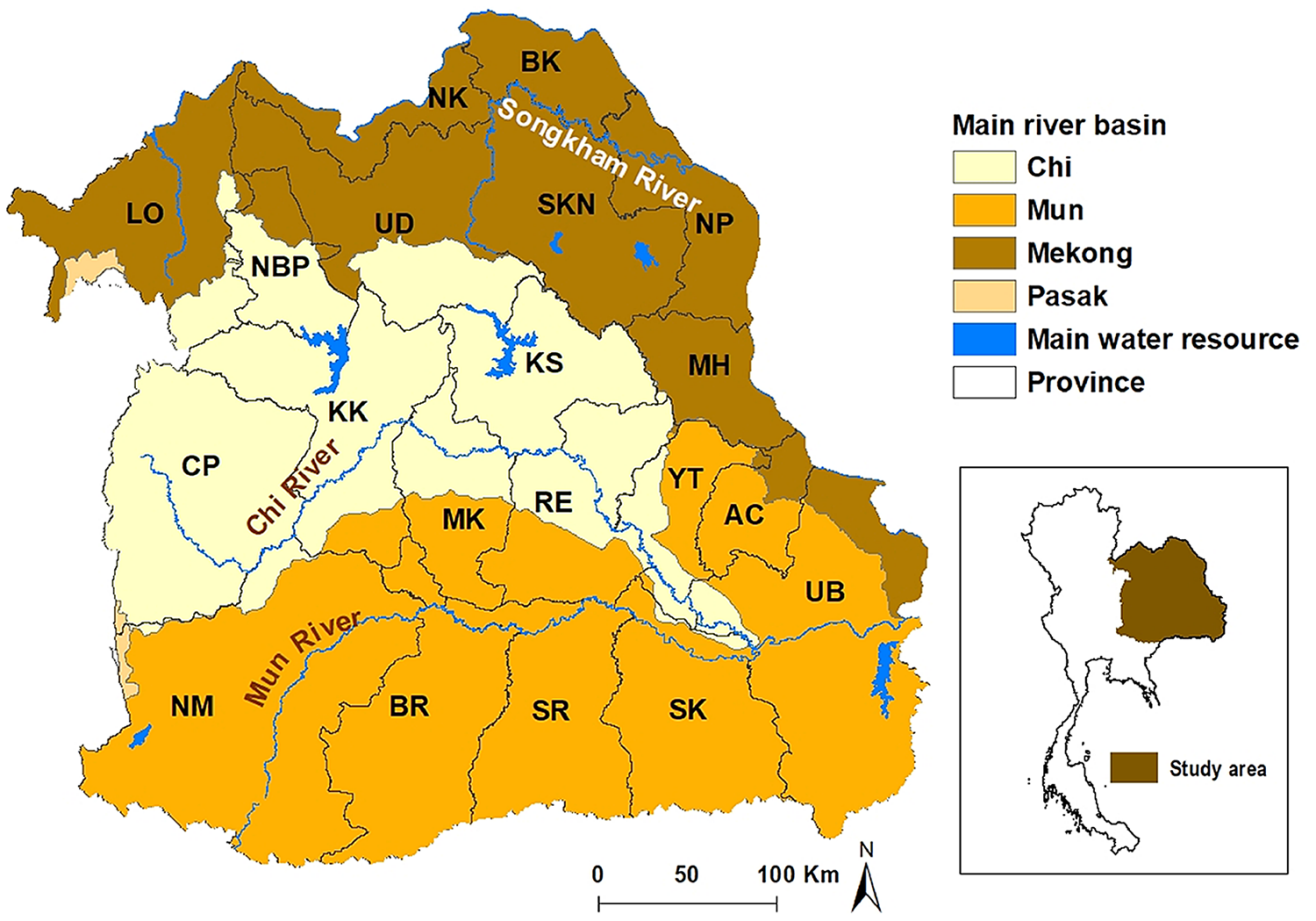
Map of study area. Provinces: *AC* Amnat Charoen, *BK* Bueng Kan, *BR* Buriram, *CP* Chaiyaphum, *KS* Kalasin, *KK* Khon Kaen, *LO* Loei, *MH* Mukdahan, *MK* Maha Sarakham, *NP* Nakhon Phanom, *NR* Nakhon Ratchasima, *NBP* Nong Bua Lamphu, *NK* Nong Khai, *RE* Roi Et, *SKN* Sakon Nakhon, *SK* Sisaket, *SR* Surin, *UB* Ubon Ratchathani, *UD* Udon Thani, *YT* Yasothon. Maps were created using ArcGIS software version 10.5.1 (ESRI: https://www.esri.com/en-us/home).

Northeast Thailand is located between latitudes 14.50° N and 17.50° N, and between longitudes 102.12° E and 104.90° E and covers an area of approximately 168,854 km^2^ (Fig. 1). There are 3 main river basins in this region; the Chi, Mun, and Mekong. Northeast Thailand is divided into 20 provinces (Fig. 1). In this study, we utilize data from all 2,678 sub-districts of these 20 provinces.

### Study population

A CCA screening program, the Cholangiocarcinoma Screening and Care Program (CASCAP), has been underway in Northeast Thailand since 2013. The target population for CASCAP includes all residents of Northeast Thailand aged ≥ 40 years. The program aims to screen all participants for *O. viverrini* infection and hepatobiliary abnormalities associated with CCA and to provide confirmatory diagnosis of suspected cases of CCA.

The CASCAP study mainly recruits participants through nine tertiary care hospitals in Northeast Thailand, as well as through mobile screening clinics in rural areas. Participants are recruited in these settings in two main ways. Firstly, patients attending these participating institutions for any reason and who had not displayed any symptoms of CCA were invited to join the program and receive initial screening. This is the ‘screening’ group. The second ‘walk-in’ group comprised people who attended the hospital with symptoms indicating CCA. This study includes both the walk-in and screening groups.

The study population for this study are all CASCAP participants who received ultrasound (US) screening for LM, PDF, and BDD, between February 2013 and December 2017. Detailed recruitment details for the CASCAP study have been published elsewhere^20^. Individual-level data for these patients were aggregated at the sub-district level, based on the CASCAP database. Sub-district populations for the study period (2013–2017) were obtained from the Official Statistics Registration Systems of Thailand website (https://stat.bora.dopa.go.th/new_stat/webPage/statByYear.php).

### Outcome and independent variables

The main outcome variables for this study were LM, PDF, and BDD identified through US screening by either radiologists or well-trained general practitioners and verified centrally by a specialist radiologist at a tertiary hospital. ‘All hepatobiliary abnormality’ was defined as identification of any of LM, PDF, BDD, fatty liver disease, cirrhosis and parenchymal change, while a sub-set of these conditions comprising LM, PDF, and BDD was defined as ‘hepatobiliary abnormalities associated with CCA’. A questionnaire completed by participants collected further independent variables including sex and age at enrollment and history of *O. viverrini* infection.

### Statistical analysis

The prevalence of US findings (LM, PDF, and BDD) in percentages were calculated by using the number of abnormal cases as the numerator and the total number of US participants as the denominator. All analyses were performed using STATA version 15 (StataCorp, College Station, TX, USA).

We adjusted for the varying population sizes by calculating Standardized Morbidity Ratios (SMR) for overall abnormalities, LM, PDF, and BDD, for each sub-district in Northeast Thailand for 2013–2017 using the formula:

$${Y}_{i}= \frac{{O}_{i}}{{E}_{i}}$$

where *Y*_*i*_ is the SMR in sub-district *i*, *O*_*i*_ is the observed number of cases of each outcome (overall abnormalities, LM, PDF, and BDD) in the sub-district and *E*_*i*_ is the expected number of cases in the sub-district across the study period. The expected number of cases was calculated by multiplying the population of each sub-district by the overall crude rates of these abnormalities during the study period^21^.

### Spatial analysis

The region studied in this analysis encompassed 2678 sub-districts in Northeast Thailand. The administrative boundaries for each of these sub-districts were defined by polygon shapefiles, sourced from the DIVA-GIS website (www.diva-gis.org). The spatial datasets including SMR of overall abnormalities, PDF, LM, and BDD were imported into the ArcGIS 10.5.1 (ESRI Inc. Redlands, CA, USA) and projected to the Universal Transverse Mercator (UTM) coordinate system zone 48 N.

MODIS data products including land surface temperature (LST), normalized difference vegetation index (NDVI) and normalized difference water index (NDWI) were downloaded from the United States Geological Survey (USGS) EROS Data Center^21^. Environmental data were collated from 1 January 2001 through 31 December 2017. Resolution was set at 0.25 km^2^ and data were processed to produce a mosaic covering the study area in Northeast Thailand using ArcGIS 10.5.1. Altitude data were obtained from the WorldClim database (www.worldclim.org). Polyline shapefiles for streams and rivers in Northeast Thailand were obtained from the DIVA-GIS website. The administrative boundary map included 2678 sub-district level areas^22^.

We defined LM + PDF + BDD cases using the collective term LPB. The spatial datasets, including demographic and environmental data, as well as LPB cases were joined to the sub-district boundary map of Northeast Thailand to extract the spatial values for statistical analyses.

A preliminary bivariate Poisson regression measuring associations between LPB cases with dependent variables (age, sex, NDVI, NDWI, LST, altitude, distance to water resource, and history of *O.viverrini* infection) was undertaken to identify significant covariates. Age, sex, distance to water resource, and history of *O. viverrini* infection were the only variables with significant associations with LBP prevalence (*P* value < 0.05) in the bivariate models and were included in the final models. Finally, Pearson correlation analyses were conducted to assess collinearity among all the included variables. All the preliminary statistical analyses were performed using STATA software version 15.0 (Stata Corporation, College Station, TX, USA).

### Spatial Poisson regression analysis

A zero-inflated Poisson (ZIP) regression model was selected over a standard Poission regression because 98.82% of data points were zeros (i.e. a majority of sub-districts had zero cases for the outcome). In addition, ZIP regression had a better fit than the Poisson regression, as indicated by a lower AIC and BIC. ZIP regression was undertaken using a Bayesian framework in the WinBUGS software version 1.4.3 (Medical Research Council, Cambridge, UK). Three models were built to incorporate spatial, non-spatial and a combination of both spatial and non-spatial random effects. The first model (Model I) included age, sex, distance to water resource, and history of *O. viverrini* infection as unstructured random effects. The Model II was based on the spatially structured random effects. The final model (Model III), a convolution model, included the same covariates as the preceding two models, and both spatially structured and unstructured random effects.

The convolution model, with an outcome of observed cases of LPB (numbers), Y, for the *i*th sub-district in Northeast Thailand (i = 1 to 2678), for the *j*th age group and *k*th sex group was structured as follows:

$$P({Y}_{ijk}= {y}_{ijkl})=\left\{\begin{array}{l}\omega +1 \left(1-\omega \right){e}^{-\mu }, {y}_{ijk}=0\\ \left(1-\omega \right){e}^{-\mu } {\mu }_{ijk}^{{y}_{ijk}}/{y}_{ijk}, {y}_{ijk}>0;\end{array}\right.$$

$$\begin{gathered} Y_{{ijk}} ~\sim {\text{Poisson}}(\mu _{{ijk}} ) \hfill \\ {\text{log}}(\mu _{{ijk}} ) = {\text{log}}({\text{E}}_{{ijk}} ){\text{ }} + \theta _{{ijk}} \hfill \\ \theta _{{ijk}} = \alpha + \beta _{1} \times {\text{Age}}_{j} + \beta _{2} \times {\text{ sex}}_{k} + \beta _{3} \times {\text{distance to water resource}}_{i} + \beta _{4} \times {\text{history of }}O.\;viverrini\;{\text{infection}}_{i} + {\text{u}}_{i} + {\text{s}}_{i} \hfill \\ \end{gathered}$$

where E_*ijk*_ is the expected number of LPB cases (offsetting population size) in sub-district *i*, age group *j*, sex group *k* and θ_*ijk*_ is the mean log relative risk (RR); α is the intercept, and *β*_1_*, β*_2_*, β*_3_*,* and *β*_4_ are the coefficients for age (≤ 60 years as the reference category), sex (female as the reference category), distance to water resource (in kilometres), and history of *O. viverrini* infection; u_*i*_ and s_*i*_ are the unstructured and structured random effects (assuming a mean of zero and variances of σ_u_^2^ and σ_s_^2^ respectively).

In modelling the spatially structured random effects, a conditional autoregressive (CAR) prior structure was used. Spatial relationships between sub-districts within the study area were modelled using an adjacency weights matrix. If sub-districts shared a border, a weight of 1 was used while 0 was assigned if they did not. A flat prior distribution was used for the intercept and a normal prior distribution was used for the coefficients (with a mean of zero and a precision, the inverse of variance, of 0.0001). The precision of the unstructured and spatially structured random effects was modelled using non-informative gamma distributions with shape and scale parameters of 0.001. These methods have previously been used in spatial analysis of cholangiocarcinoma in Northeast Thailand^23^.

Before storing values from the posterior distributions of variables, a burn-in of 10,000 iterations was discarded. Subsequent sets of 15,000 iterations were run and checked for evidence of convergence of the Monte Carlo chains. We repeated these sets of 15,000 iterations three times, and discarded the results. At this stage we visually examined the posterior density plots and determined convergence had been achieved. The resulting model had the lowest deviance information criterion (DIC), and was chosen based on finding a balance between fit and parsimony. Statistical significance of the covariates was defined by an α-level of 0.05, which was calculated using the 95% credible intervals (95% CrI) for relative risks (RR) (signifcant variables had a 95%CrI for the RR that excluded 1). ArcGIS 10.5.1 software was used to generate maps of the spatial distribution of posterior means of the unstructured and structured random effects.

### Ethical considerations

The study was approved by Khon Kaen University Ethics Committee for Human Research (HE551404 and HE621288). All methods were carried out in accordance with relevant guidelines and regulations. All participants gave written informed consent to participate in the study and for their anonymized data to be used for statistical analysis and dissemination.

## Results

### Descriptive statistics

A total of 357,203 people underwent US screening. Approximately two-thirds of all partipants were female (61.3%) and around three quarters (74%) were aged between 40 and 60 years old, with a mean age of 54.5 years (SD 9.5). The overall prevalence of any hepatobiliary abnormalities was 38.7% (*n* = 138,236). Stratified by sex and age at enrolment, PDF was the most common hepatobiliary abnormality in males for both age groups: ≤ 60 years 20.1% (19,114) and > 60 years old 22.0% (*n* = 9,258). While amongst females, the most common hepatobiliary abnormality was FLD for both age groups: ≤ 60 years, 20.9% (*n* = 34,834) and > 60 years 19.6% (*n* = 9,883) (Table 1). There were statistically significant differences in the prevalence of hepatobiliary abnormalities between sex and age groups with males and those aged over 60 having the highest prevalence (Table 2).

**Table 1:** Prevalence of hepatobiliary abnormalities stratified by sex and age at enrolment.

Characteristics	Subjects	All*		FLD		CIR		PCC		LM		PDF		BDD	
		*n*	(%)	*n*	(%)	*n*	(%)	*n*	(%)	*n*	(%)	*n*	(%)	*n*	(%)
Over all	357,203	138,236	(38.7)	68,451	(19.2)	1802	(0.5)	4281	(1.2)	7033	(2.0)	67,128	(18.8)	4276	(1.2)
**Male**															
≤ 60	95,114	37,495	(39.4)	17,228	(18.1)	903	(1.0)	1393	(1.5)	1609	(1.7)	19,114	(20.1)	1141	(1.2)
> 60	42,051	16,742	(39.8)	5994	(14.3)	417	(1.0)	981	(2.3)	1,350	(3.2)	9,258	(22.0)	1242	(3.0)
**Female**															
≤ 60	166,866	63,402	(38.0)	34,834	(20.9)	288	(0.2)	1249	(0.8)	2786	(1.7)	29,241	(17.5)	145	(0.6)
> 60	50,544	19,572	(38.7)	9883	(19.6)	182	(0.4)	644	(1.3)	1248	(2.5)	9020	(17.9)	821	(1.6)

*FLD* fatty liver disease, *CIR* cirrhosis, *PCC* parenchymal change, *LM* liver mass, *PDF* periductal fibrosis, *BDD* bile duct dilatation, *n* number of abnormality cases.

*All hepatobiliary abnormality was defined as people who have symptoms from any of the outcomes LM, PDF, BDD, FLD, CIR or PCC.

**Table 2:** Prevalence of any hepatobiliary abnormality by sex and age at enrollment.

Characteristics	Subjects	Abnormalities		*P* value*
		*n*	(%)	
**Sex**				< 0.001
Female	219,034	83,602	(38.2)	
Male	138,128	54,617	(39.5)	
**Age (years)**				< 0.001
≤ 60	261,993	100,902	(38.5)	
> 60	92,597	36,315	(39.2)	

*P value from chi-square test.

Table 3 shows only the hepatobiliary abnormalities that are associated with CCA. Twenty-one percent (*n* = 75,640) of study participants had abnormalities that are associated with CCA. When stratified by sex and age group, for male ≤ 60 years, the commonest abnormalities were a combination of PDF and BDD, 0.4% (*n* = 341) followed by LM and PDF, 0.3% (*n* = 277). However, for males aged > 60 years, combinations of LM and BDD (0.7%, *n* = 273) and PDF and BDD, (0.7%, *n* = 275) were the most common. For females of both age groups, LM and PDF were the most common abnormalities; 0.3% (*n* = 422) and 0.4% (*n* = 187), respectively. There was a statistically significant difference between men and women, and age groups for overall abnormalities that cause CCA (Table 4).

**Table 3:** Distribution of hepatobiliary abnormalities biologically associated with CCA, and combinations of hepatobiliary abnormalities, stratified by sex and age at enrolment.

Characteristics	Subjects	All*		LM + PDF		LM + BDD		PDF + BDD		LM + PDF + BDD (LPB)	
		*n*	(%)	*n*	(%)	*n*	(%)	*N*	(%)	*n*	(%)
Over all	357,203	75,640	(21.2)	1,127	(0.3)	737	(0.2)	1,041	(0.3)	108	(0.03)
**Male**											
≤ 60	95,114	21,101	(22.2)	277	(0.3)	169	(0.2)	341	(0.4)	24	(0.0)
> 60	42,051	11,111	(26.4)	233	(0.6)	273	(0.7)	275	(0.7)	42	(0.1)
**Female**											
≤ 60	166,866	32,266	(19.3)	422	(0.3)	121	(0.1)	285	(0.2)	22	(0.0)
> 60	50,544	10,615	(21.0)	187	(0.4)	171	(0.3)	136	(0.3)	20	(0.0)

*LM* liver mass, *PDF* periductal fibrosis, *BDD* bile duct dilatation, *n* number of abnormality cases.

*All hepatobiliary abnormalities was defined as people who have symptoms from any of the outcomes (LM, PDF, and BDD).

**Table 4:** Prevalence of hepatobiliary abnormalities associated with cholagiocarcinoma by sex and age at enrollment.

Characteristics	Subjects	Abnormalities associated with CCA		*P* value*
		*n*	(%)	
**Sex**				< 0.001
Female	219,034	43,197	(19.7)	
Male	138,128	32,436	(23.5)	
**Age (years)**				< 0.001
≤ 60	261,993	53,371	(20.4)	
> 60	92,597	21,726	(23.5)	

*CCA* cholagiocarcinoma.

*P value from chi-square test.

Supplementary Figure S1 shows the SMR for the distribution of hepatobiliary abnormalities in the Northeast region of Thailand. The map reveals that the highest SMR of overall hepatobiliary abnormalities was found in Amnat Charoen, Yasothon, Surin, Sisaket, Nakon Phanom, Bueng Kan, and Loei Provinces (Supplementary Fig. S1A). A similar distribution was found for PDF (Supplementary Fig. S1B). However, a higher burden of LM was located in Roi Et Province (Supplementary Fig. S1C), while BDD was sporadically distributed in Roi Et, Kalasin and Udon Thani Provinces (Supplementary Fig. S1D). The SMR map of overall hepatobiliary abnormalities stratified by sex, shows a similar trend with higher incidence areas in Amnat Charoen, Yasothon, Surin, Sisaket, Nakon Phanom, Bueng Kan, and Loei Provinces (Supplementary Fig. S2).

**Figure 2:**
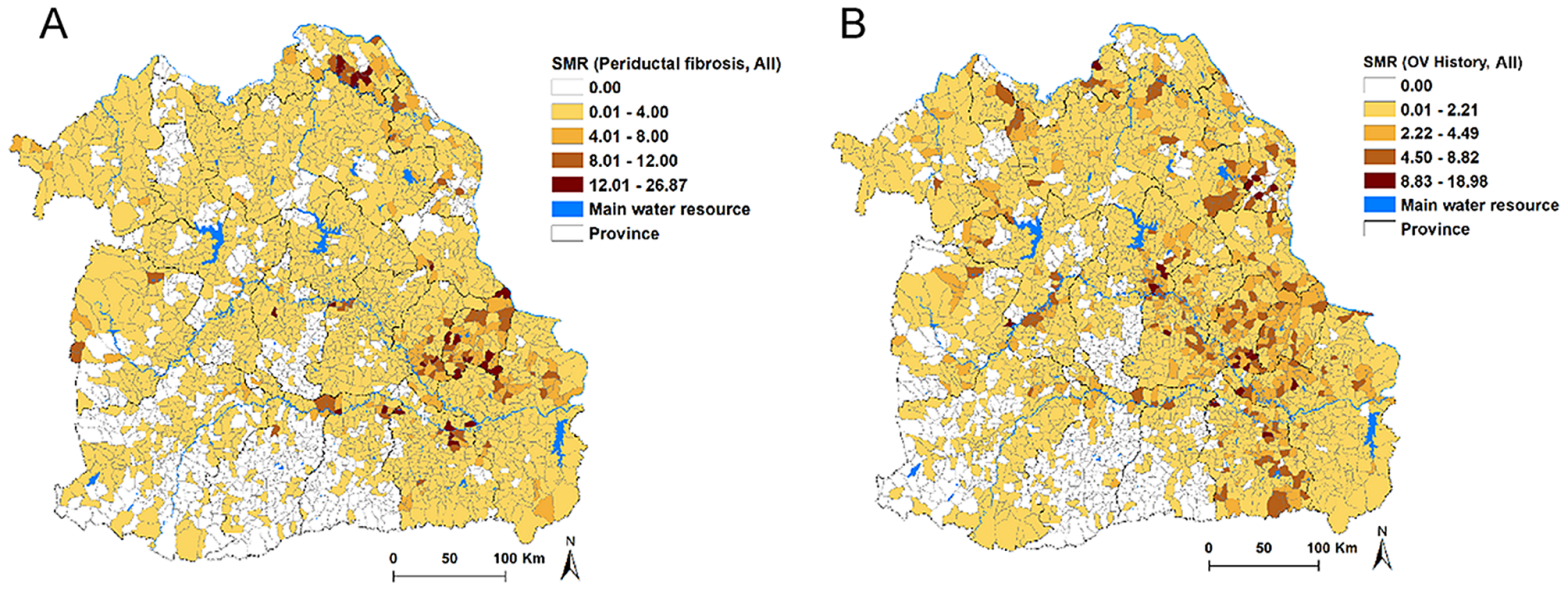
Standardized morbidity ratio of: (**A**) periductal fibrosis and (**B**) *Opisthorchis viverrini* infection history. *OV* *Opisthorchis viverrini*. Maps were created using ArcGIS software version 10.5.1 (ESRI: https://www.esri.com/en-us/home).

There was an overlap in the SMR distribution for PDF (Fig. 2A) and *O. viverrini* infection history (Fig. 2B) with higher incidence areas in Amnat Charoen, Yasothon, Surin, Sisaket, Nakon Phanom, and Bueng Kan Provinces.

### Spatial Poisson regression analysis

**Figure 3:**
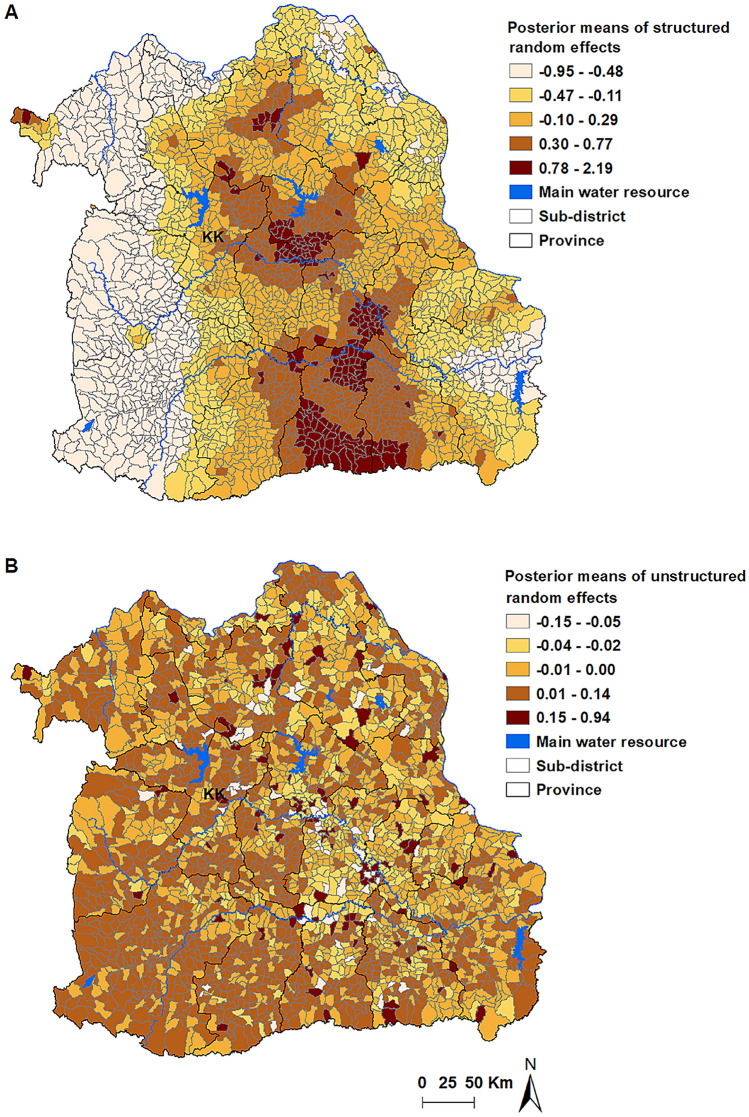
Spatial distributions of the posterior means of random effects for LPB in Northeast Thailand in Model III. (**A**) Spatially structured random effects (**B**) unstructured random effects. *KK* Khon Kaen Province. Maps were created using ArcGIS software version 10.5.1 (ESRI: https://www.esri.com/en-us/home).

Table 5 shows the Bayesian spatial and non-spatial models for LPB. According to the value of the DIC statistic, Model III that included both unstructured and structured random effects was the best-fitting. In Model III, age and sex were significantly associated with LPB cases. LPB diagnosis was more common in participants aged more than 60 years (RR 3.51, 95% CrI 2.39–5.27). LBP was also more common among males than females (RR 2.26, 95% CrI 1.53–3.40). Spatial clustring was demonstrated after accounting for the model covariates, shown in the map of the posterior means of the spatially structured random effects (Fig. 3). Surin, Sisaket, Roi Et, Kalasin, and Maha Sarakham Provinces, which located in the Mun and Chi River Basins, were associated with clusters of LPB. Songkham River Basin province of Udon Thani was contained some high risk sub-districts (Fig. 3A). The unstructured random effects showed a random spatial pattern as expected (Fig. 3B).

**Table 5:** Regression coefficients, RRs and 95% credible interval from Bayesian models for LPB in Northeast Thailand.

Model/variables	Coefficient, posterior mean	95% CrI	RR, posterior mean	95% CrI
**Model I (unstructured)**				
α (Intercept)	− 9.36	− 10.10, − 8.63		
Age*	1.26	0.88, 1.66	3.54	2.40, 5.24
Sex**	0.81	0.42, 1.21	2.25	1.53, 3.34
Distance to water resource (Km)	0.06	− 0.14, 0.24	1.06	0.87, 1.26
History of *O. viverrini* infection	− 0.16	− 0.76, 0.46	0.85	0.47, 1.59
Heterogeneity				
Structured (variance)				
Unstructured (variance)	1.81	0.52, 11.36		
DIC	1061.15			
**Model II (structured)**				
α (Intercept)	− 9.47	− 10.23, − 8.80		
Age*	1.25	0.86, 1.65	3.48	2.36, 5.20
Sex**	0.82	0.43, 1.21	2.26	1.54, 3.35
Distance to water resource (Km)	0.05	− 0.15, 0.22	1.04	0.86, 1.23
History of *O. viverrini* infection	− 0.11	− 0.70, 0.55	0.90	0.50, 1.73
Heterogeneity				
Structured (variance)	0.45	0.24, 0.91		
Unstructured (variance)				
DIC	1045.15			
Model III (structured and unstructured)***				
α (Intercept)	− 9.61	− 10.39, − 8.91		
Age*	1.26	0.87, 1.66	3.51	2.39, 5.27
Sex**	0.81	0.42, 1.23	2.26	1.53, 3.40
Distance to water resource (Km)	0.05	− 0.15, 0.24	1.05	0.86, 1.26
History of *O. viverrini* infection	− 0.12	− 0.73, 0.51	0.89	0.48, 1.67
Heterogeneity				
Structured (variance)	0.58	0.18, 1.86		
Unstructured (variance)	16.10	0.97, 203.90		
DIC	1036.28			

*CrI* credible intervals, *RR* relative risks, *DIC* deviance information, *Km* kilometres.

*Age ≤ 60 years was reference; **female sex was reference; ***best fit model.

### Discussion

This is the first study to present a spatial analysis of hepatobiliary abnormalities, which are amongst the leading health problems in Northeast Thailand due to their association with the development of cholangiocarcinoma (CCA). Overall, nearly 40% of those screened had some form of hepatobiliary disorder indicating the magnitude of the problem. Spatial analysis also identified particular risk areas in upper and lower Northeast Thailand.

In this study, sex and age differences in the prevalence of hepatobiliary abnormalities biologically associated with CCA were noted. Prevalence of abnormalities was significantly higher in males than females, and higher in older than younger participants. These sex differences have also been observed in previous research on hepatobiliary abnormalities in opisthorchiasis-endemic areas of Northeast Thailand, where a significantly higher prevalence of advanced periductal fibrosis was found in males compared to females. These abnormalities were significantly associated with intensity of *O. viverrini* infection^19,24^, a causative factor in the development of these hepatobiliary abnormalities. Males are known to be at higher risk of *O. viverrini* infection than females, as a result of their behavior related to eating raw cyprinid fish, alcohol consumption and smoking, and this leads to higher hepatobiliary abnormalities and CCA rates^25,26^. The reasons for the significantly increased hepatobiliary abnormality levels in older age groups in this region are not yet fully understood. Many factors have been proposed such as chronic inflammation, accumulated genetic alterations, immune response and cumulative past exposure to *O. viverrini* infection^25,27–29^.

In this study, Bayesian spatial analysis was applied to verify the existence of spatial clusters for hepatobiliary abnormalities. The results showed that there is relatively large geographical variation in prevalence between areas of Northeast Thailand, manifesting in large geographical clusters. The main clusters of hepatobiliary abnormalities are located along the Chi, Mun, and Songkram Rivers. These rivers are opisthorchiasis-endemic areas which obtain flows from the Mekong River and its tributaries, creating water resources, such as lakes, marshes, and ponds, in the wetland plains, habitats for the intermediate hosts, snails and fish^18,30,31^.

Many previous community-based ultrasound studies in opisthorchiasis-endemic areas in Northeast Thailand suggest that hepatobiliary abnormalities and increased periportal fibrosis are common in people with chronic opisthorchiasis^19,24,32^. *Opisthorchis viverrini* infection risk is also significantly related to the distance to the nearest fresh water bodies. A study in southern Lao PDR showed that the risk of *O. viverrini* infection was found to be higher for people living near freshwater bodies^33^. People living in close proximity to rivers or other fresh water bodies were more likely to incur *O. viverrini* infection by eating raw or undercook cyprinid fish compared to people living further from the water.

This study has some limitations. The sample used for the analysis of hepatobiliary abnormalities is large, with over 350,000 participants screened, but is not representative of the general population of Thailand. Also, participant recruitment through health care facilities, and their willingness to participate, may indicate some underlying differences from the overall population, resulting in potential bias. It is likely that given these two limitations, the actual prevalence of hepatobiliary abnormalities in the general population will be lower than that estimated here. In particular, the study sample only includes those aged 40 years and older. A further limitation is that the *O. viverrini* infection history measurement was self-reported, leading to possibile recall bias. Finally, given that hepatobiliary abnormalities have a long lead time there is some possibility that population mobility may introduce some bias in our results. That is, some participants diagnosed in this study may have in fact moved into the study area when already affected, or have been exposed to risk factors outside our study area. However, given that Northeast Thailand has the highest prevalence of hepatobiliary abnormalities in the country, in-migration would have the effect of lowering prevalence rates.

The identification of several significant clusters of hepatobiliary abnormalities in this study is important for informing resource allocation in Northeast Thailand. This approach is novel in that it identifies clusters of hepatobiliary abnormalities, which are contributing factors to the development of CCA, and relates them to the main aetiological cause in Northeast Thailand (*O. viverrini* infection). These findings may be used not only to direct ongoing and future outreach and public education efforts, but also have policy implications within the area of service assessment and resource allocation. In particular, the identification of a high prevalence of hepatobiliary abnormalities in a previously unscreened population may indicate barriers to obtaining preventative screening in this region. In fact, to date almost all control programs for opisthorchiasis and cholangiocarcinoma in northeast Thailand have focused only on primary prevention, screening for *O. viverrini* infection. Only with the CASCAP program has this attention extended to secondary prevention, screening for CCA and periductal fibrosis, and patient treatment methodologies, in the tertiary hospital and clinics^20,34^. The high prevalences found here have important implications for the ability to identify patients at risk of development of CCA, a disease with very low survival rates.

### Conclusions

Spatial clusters of hepatobiliary abnormalities were found in in Northeast Thailand. Knowledge about these spatial patterns can provide useful information to policymakers in the targeting of of screening for CCA in the targeted population and resourcing of healthcare facilities to provide better treatment for CCA patients.

## Supplementary information

Supplementary Figures

## Data Availability

The datasets generated during and/or analyzed during the current study are available from the corresponding author on reasonable request.

## References

[1] Moore MA (2010). Cancer epidemiology in mainland South-East Asia—past, present and future. Asian Pac. J. Cancer Prev..

[2] Sripa B, Pairojkul C (2008). Cholangiocarcinoma: Lessons from Thailand. Curr. Opin. Gastroenterol..

[3] Srivatanakul P, Sriplung H, Deerasamee S (2004). Epidemiology of liver cancer: An overview. Asian Pac. J. Cancer Prev..

[4] Kubo S (2015). Screening and surveillance for occupational cholangiocarcinoma in workers exposed to organic solvents. Surg. Today.

[5] Fontan FJ, Reboredo AR, Siso AR (2015). Accuracy of contrast-enhanced ultrasound in the diagnosis of bile duct obstruction. Ultrasound Int. Open.

[6] Songserm N (2012). Risk factors for cholangiocarcinoma in high-risk area of Thailand: Role of lifestyle, diet and methylenetetrahydrofolate reductase polymorphisms. Cancer Epidemiol..

[7] Tao LY (2010). Risk factors for intrahepatic and extrahepatic cholangiocarcinoma: A case–control study in China. Liver Int..

[8] Welzel TM (2007). Risk factors for intrahepatic and extrahepatic cholangiocarcinoma in the United States: A population-based case–control study. Clin. Gastroenterol. Hepatol..

[9] Shaib YH (2007). Risk factors for intrahepatic and extrahepatic cholangiocarcinoma: A hospital-based case–control study. Am. J. Gastroenterol..

[10] Shaib YH, El-Serag HB, Davila JA, Morgan R, McGlynn KA (2005). Risk factors of intrahepatic cholangiocarcinoma in the United States: A case–control study. Gastroenterology.

[11] West J, Wood H, Logan RF, Quinn M, Aithal GP (2006). Trends in the incidence of primary liver and biliary tract cancers in England and Wales 1971–2001. Br. J. Cancer.

[12] National Cancer Institue. *Guidelines for Screening, Diagnosis and Treatment of Liver Cancer and Cholangiocarcinoma*. 81 (National Office of Buddhism, 2011).

[13] Khan SA (2012). Guidelines for the diagnosis and treatment of cholangiocarcinoma: An update. Gut.

[14] Maetani Y (2001). MR imaging of intrahepatic cholangiocarcinoma with pathologic correlation. Am. J. Roentgenol..

[15] Chamadol N (2014). Imaging in Cholangiocarcinoma.

[16] Xu HX (2012). Contrast-enhanced ultrasound of intrahepatic cholangiocarcinoma: Correlation with pathological examination. Br. J. Radiol..

[17] Chamadol N (2014). Histological confirmation of periductal fibrosis from ultrasound diagnosis in cholangiocarcinoma patients. J. Hepatobil. Pancreat. Sci..

[18] Suwannatrai A, Saichua P, Haswell M (2018). Epidemiology of *Opisthorchis viverrini* infection. Adv. Parasitol..

[19] Mairiang E (2012). Ultrasonography assessment of hepatobiliary abnormalities in 3359 subjects with *Opisthorchis viverrini* infection in endemic areas of Thailand. Parasitol. Int..

[20] Khuntikeo N (2015). Cohort profile: Cholangiocarcinoma screening and care program (CASCAP). BMC Cancer.

[21] Alene KA, Viney K, McBryde ES, Clements AC (2017). Spatial patterns of multidrug resistant tuberculosis and relationships to socio-economic, demographic and household factors in northwest Ethiopia. PLoS One.

[22] United States Geological Survey. *Earth Resources Observation and Science (EROS) Center*. https://www.usgs.gov/centers/eros (2020).

[23] Suwannatrai AT (2019). Bayesian spatial analysis of cholangiocarcinoma in Northeast Thailand. Sci. Rep..

[24] Elkins DB (1996). Cross-sectional patterns of hepatobiliary abnormalities and possible precursor conditions of cholangiocarcinoma associated with *Opisthorchis viverrini* infection in humans. Am. J. Trop. Med. Hyg..

[25] Honjo S (2005). Genetic and environmental determinants of risk for cholangiocarcinoma via Opisthorchis viverrini in a densely infested area in Nakhon Phanom, northeast Thailand. Int. J. Cancer.

[26] Steele JA (2018). Thinking beyond Opisthorchis viverrini for risk of cholangiocarcinoma in the lower Mekong region: A systematic review and meta-analysis. Infect. Dis. Poverty.

[27] Sripa B (2011). Opisthorchiasis and Opisthorchis-associated cholangiocarcinoma in Thailand and Laos. Acta Trop..

[28] Sithithaworn P, Yongvanit P, Duenngai K, Kiatsopit N, Pairojkul C (2014). Roles of liver fluke infection as risk factor for cholangiocarcinoma. J. Hepatobil. Pancreat. Sci..

[29] Haswell-Elkins MR (1991). Immune responsiveness and parasite-specific antibody levels in human hepatobiliary disease associated with *Opisthorchis viverrini* infection. Clin. Exp. Immunol..

[30] Upatham ES (1988). A review of experimental and field research on the human liver fluke, *Opisthorchis viverrini*. J. Sci. Soc.Thai..

[31] Sripa B (2007). Liver fluke induces cholangiocarcinoma. PLoS Med..

[32] Sripa B (2009). Advanced periductal fibrosis from infection with the carcinogenic human liver fluke *Opisthorchis viverrini* correlates with elevated levels of interleukin-6. Hepatology.

[33] Forrer A (2012). Spatial distribution of, and risk factors for, *Opisthorchis viverrini* infection in southern Lao PDR. PLoS Negl. Trop. Dis..

[34] Khuntikeo N (2016). Comprehensive public health conceptual framework and strategy to effectively combat cholangiocarcinoma in Thailand. PLoS Negl. Trop. Dis..

