# Supplementary material for: Spatial analysis of hepatobiliary abnormalities in a population at high-risk of cholangiocarcinoma in Thailand
Source: Sci Rep. 2020 Oct 8;10:16855. doi: 10.1038/s41598-020-73771-0 (PMC7545164; doi:10.1038/s41598-020-73771-0)
Supplement: Supplementary file 1 — Supplementary Figures [file 41598_2020_73771_MOESM1_ESM.docx]

**Spatial analysis of hepatobiliary abnormalities in a population at high-risk of cholangiocarcinoma in Thailand**

Kavin Thinkhamrop^1,2,3^, Apiporn T. Suwannatrai^3,4^*, Nittaya Chamadol^1,5^, Narong Khuntikeo^1,6^, Bandit Thinkhamrop^1,2,7^, Pongdech Sarakarn^3,7^, Darren J. Gray^8^, Kinley Wangdi^8^, Archie C. A. Clements^9,10^, and Matthew Kelly^8^

^1^Cholangiocarcinoma Screening and Care Program (CASCAP), Faculty of Medicine, Khon Kaen University, Khon Kaen, 40002, Thailand

^2^Data Management and Statistical Analysis Center (DAMASAC), Faculty of Public Health, Khon Kaen University, Thailand

^3^Health and Epidemiology Geoinformatics Research (HEGER), Faculty of Public Health, Khon Kaen University, Thailand

^4^Department of Parasitology, Faculty of Medicine, Khon Kaen University, Thailand ^5^Department of Radiology, Faculty of Medicine, Khon Kaen University, Thailand

^6^Department of Surgery, Faculty of Medicine, Khon Kaen University, Thailand

^7^Epidemiology and Biostatistics Section, Faculty of Public Health, Khon Kaen University, Thailand

^8^Department of Global Health, Research School of Population Health, Australian National University, Canberra, Australia.

^9^Faculty of Health Sciences, Curtin University, Bentley, Western Australia, Australia.

^10^Telethon Kids Insitute, Nedlands, Western Australia, Australia

Correspondence and requests for materials should be addressed to A.T.S. (e-mail: apiporn@kku.ac.th)

**Supplementary Figure**

**
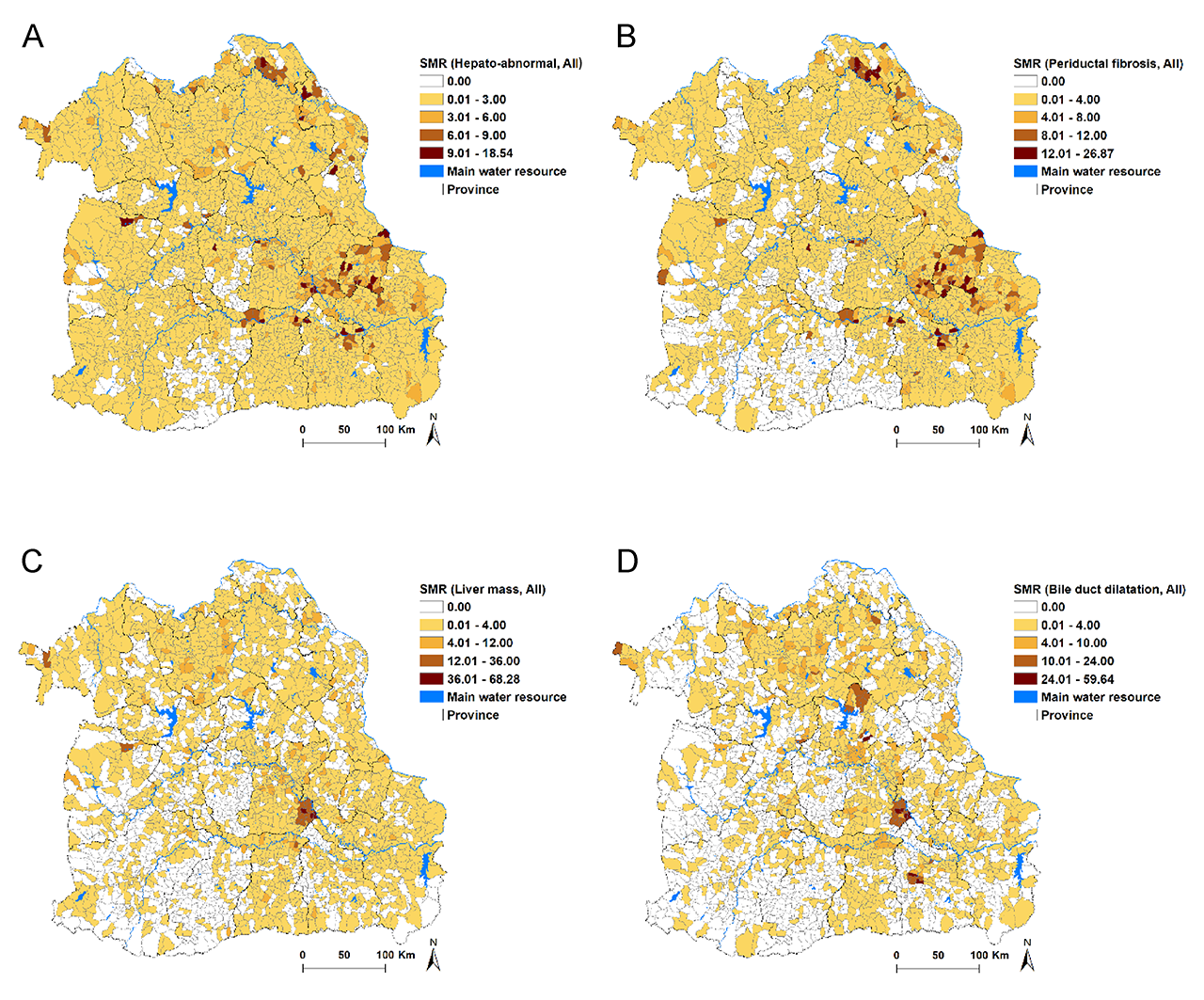
**

**Supplementary Figure 1** Standardize morbidity ratio of overall hepatobiliary abnormalities (A), periductal fibrosis (B), liver mass (C), and bile duct dilatation (D). Maps were created using ArcGIS software version 10.5.1 (ESRI: <https://www.esri.com/en-us/home>)


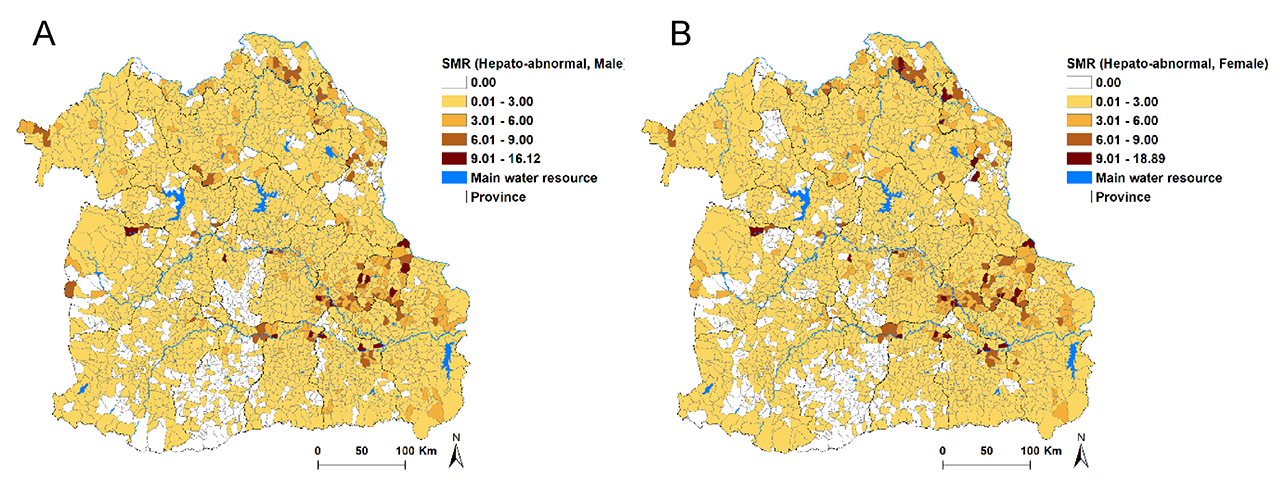


**Supplementary Figure 2** Standardize morbidity ratio of overall hepatobiliary abnormalities in male (A), and female (B). Maps were created using ArcGIS software version 10.5.1 (ESRI: <https://www.esri.com/en-us/home>)
